# Non-targeted effects and DNA: vulnerabilities at the interface

**DOI:** 10.3389/fonc.2026.1952660

**Published:** 2026-10-16

**Authors:** Carmel Mothersill, Andrej Rusin, Colin Seymour

**Affiliations:** Department of Biology, McMaster University, Hamilton, ON, Canada

**Keywords:** bystander effects, DNA, genomic instability, lethal mutations, non-targeted effects, radiation quality effects, repair of DNA damage

## Abstract

**Objectives:**

This paper aims to explore areas of radiobiological research where DNA-targeted effects interface with non-DNA-targeted effects (NTEs). While target theory established in the 1930s claims that DNA was a primary target for radiation-induced cellular damage, non-targeted effects, although known from 1905, were largely ignored. In this narrative review, the data will be examined showing that DNA damage can not only be directly caused by radiation but also be induced in non-hit cells due to signaling factors. Similarly, DNA repair systems can be induced in situations where no radiation was delivered to the system. The implications of these data for radiotherapy, especially new modalities, and low-dose radiation protection are discussed.

**Conclusions:**

The data clearly show that out-of-field and abscopal effects as well as inter-individual signaling occur and induce a variety of responses in non-targeted cells. In many cases, these mechanisms appear to be system-level effects coordinating responses to DNA damage at a level higher and more complex than the individual cell/tissue that receives a direct radiation exposure. This implies that NTEs, although often measured using endpoints seen as “bad” at the level of the individual targeted cell, are actually protective, and counteract vulnerabilities in cellular-level DNA repair systems. This has implications for the design of novel therapies for cancer and may also help in understanding mechanisms of low-dose radiation carcinogenesis.

## Introduction: background to NTE, abscopal effects, and out-of-field effects—historical overview

Modern studies of radiation-induced bystander effects (RIBEs) and radiation-induced genomic instability (RIGI), now collectively regarded as non-targeted effects (NTEs), can be traced to the mid-1980s when a series of papers challenged the idea that radiation damage occurred in the first divisions after exposure. These early papers demonstrated that lethal damage occurred after many normal cell divisions. The damage manifested as lethal mutations or chromosomal aberrations in distant progeny. This history is reviewed in (1). The cellular damage could not have been carried by cells as the mutations/aberrations were lethal. They were also non-clonal, implying that the genome had become less able to eliminate or correct mistakes (2). These phenomena are now known as RIGI. The other phenomenon known as the RIBE or radiation-induced bystander response (RIBR) came to modern radiobiology following a paper in 1992 (3) showing that if cells were exposed to a very low fluence of alpha particles such that only about 30% of the cell population received a “hit” or energy deposition, sister chromatid exchanges (SCEs) could be detected in much more than 30% of the cell population. Subsequent work in the next two decades using medium transfer (4), microbeams (5), transwells (6), and whole animal models (7) established that signals produced by irradiated cells could induce effects in unexposed cells, tissues, or organisms receiving these signals. Both fields have been extensively reviewed (8–12), and it is now known that non-DNA-targeted effects or NTEs had actually been documented as far back as 1905, just a few years after radiation was first described [see (13) for a historical review]. The effects were variously called “indirect effects”, “abscopal effects”, “clastogenic effects”, or “out-of-field effects”, but all involved effects detected at sites, in space or time, which did not directly receive an energy deposition from radiation. Since the topic for discussion is vulnerabilities in DNA responses to ionizing radiation and the role of NTE in these responses, it is important to review the data concerning DNA effects in non-targeted systems.

NTEs in radiation biology emerged from observations that radiation-induced genetic damage cannot always be explained solely by direct energy deposition in the DNA of the responding cell. In brief, the traditional target theory held that biological effects arise primarily when radiation directly damages critical cellular targets, particularly nuclear DNA. However, studies of delayed genetic effects showed that irradiated cells could produce descendants exhibiting newly arising chromosomal abnormalities or reduced clonogenic survival generations after apparently normal cell divisions, giving rise to the concept of RIGI (14–16). These observations suggested that radiation can induce a persistent destabilized cellular state rather than simply producing DNA lesions that are directly inherited by daughter cells.

The modern concept of the RIBE was established, as reviewed in this report, most prominently by Nagasawa and Little (3), who observed increased SCEs in approximately 30% of cells exposed to extremely low fluences of α-particles despite fewer than 1% of cell nuclei being directly traversed. Subsequent studies demonstrated that genetic and cytotoxic effects could occur in neighboring unirradiated cells and that conditioned medium from irradiated cells could induce effects in unirradiated recipient cells, indicating that radiation responses could be transmitted through intercellular communication and soluble extracellular signals (3, 4). Further work demonstrated roles for gap-junction communication, membrane signaling, and oxidative and other stress-response pathways, while experiments subsequently showed that bystander cells could exhibit mutations and chromosomal aberrations as well as longer-term genomic instability. We will cover these findings in this review.

Together, genomic instability and bystander effects substantially broadened the classical model of radiation action. Radiation is now understood not only as a physical agent that directly damages DNA, but also as a perturbation capable of initiating signaling networks involving cell–cell communication, membrane and mitochondrial signaling, oxidative stress, inflammation, and potentially epigenetic alterations. These processes can produce biological and genetic consequences in cells that were never directly irradiated or can cause genetic abnormalities to emerge long after the initial exposure, with important implications for low-dose radiation biology, carcinogenesis, radiation protection, and radiotherapy (17–19).

Evidence that NTE can be protective rather than purely damaging has emerged primarily from studies of RIBEs and adaptive responses (ARs). In some experimental systems, signals released by irradiated cells induce stress responses in neighboring, unirradiated cells that increase their resistance to subsequent radiation or other genotoxic insults. These responses can include enhanced DNA repair, altered cell-cycle regulation, antioxidant responses, and elimination of damaged cells through apoptosis. For example, bystander signaling has been proposed to coordinate the removal of cells carrying pre-existing or radiation-induced damage, thereby potentially protecting the overall cell population from retaining genetically compromised cells (20, 21). This interpretation is particularly interesting because it suggests that a bystander response can function as a tissue-level quality-control mechanism, rather than simply propagating radiation damage.

Experimental evidence also supports an overlap between NTE signaling and the adaptive radiation response, in which a low-dose “priming” exposure produces increased resistance to a subsequent, larger “challenge” exposure. Studies have reported enhanced resistance to radiation-induced chromosome damage and other endpoints following low-dose conditioning, and some work suggests that signals from irradiated cells can transmit this protective phenotype to non-irradiated cells (22–25). The proposed mechanisms include activation of generalized stress responses, calcium signaling, reactive oxygen species, mitochondrial pathways, and DNA-repair processes (20, 21, 26). Thus, NTEs may sometimes act as an early-warning system: a small amount of cellular stress triggers coordinated protective responses before more substantial damage occurs.

Importantly, however, the evidence does not support the conclusion that all NTEs are inherently protective. Bystander signaling can produce either damaging or protective outcomes depending on dose, radiation quality, cell type, genetic background, and the nature of the endpoint being measured (27–32). Reviews of the field emphasize that ARs are not observed universally and that some experimental systems instead show radiosensitization or detrimental bystander effects (33–36). Consequently, the most defensible interpretation is that NTE signaling represents a context-dependent stress-response network capable of amplifying damage in some circumstances while promoting cellular survival, repair, or removal of damaged cells in others.

In this narrative review, we discuss some of the key findings in NTE and DNA effects. Because NTE research occupies a fairly small field compared to direct DNA effects, we have endeavored to collect and describe seminal work from approximately just before the advent of NTEs as we know them, when NTEs had been observed but not extensively elucidated, to the present day.

### Review methodology

This is essentially a personal narrative review for a special issue and focuses on the body of work from the authors’ laboratories in Ireland and Canada, contributed over almost 50 years of radiobiology research (1977–2026). The aim is to put the field of NTEs of radiation in context given that at least in the early days, the research findings challenged many of the concepts associated with mainstream DNA damage and repair radiobiology.

## Studies of DNA effects following non-targeted protocols, including medium transfer, microbeam, ultra-low exposures, and transwell techniques

The early data mostly involved measurements of chromosomal damage in cells exposed to plasma from irradiated patients, or in cell cultures of unexposed cells using one of several protocols for studying NTE listed above. As techniques to measure DNA damage evolved, evidence accumulated that DNA damage measured in directly irradiated cells could also be found in cells that were not irradiated and in distant progeny of irradiated cells (37–39). How can this happen was the question. Key experiments are discussed below. **Table 1** shows some relevant early key studies of non-targeted DNA effects.

**Table 1:** Several important and early studies of DNA effects following non-targeted protocols, which encompass medium transfer, microbeam, ultra-low exposures, and trans-well techniques.

Ref.	Year	Endpoint	Notes
(40)	1968	*Ex vivo* plasma transfer, chromosomal aberrations	Plasma from various patients receiving radiotherapy induced chromosomal aberrations in cultured lymphocytes.
(41)	1968	*Ex vivo* plasma transfer, chromosomal aberrations	Plasma from individuals undergoing total-body irradiation could induce chromosome breaks in cultured lymphocytes.
(42)	1974	Murine *in vivo*, chromosomal aberrations	Alpha source and systemic murine irradiations.
(43)	1977	*Ex vivo* plasma transfer, chromosomal aberrations	Plasma from individuals receiving cancer radiotherapy and heavily exposed atomic bomb survivors could include chromosome breaks in normal cultured human lymphocytes.
(44)	1979	Murine *in vivo*, SCE	Rat bone marrow cells. Alpha source, radon inhalation.
(3)	1992	Sister chromatid exchanges (SCE)	Chinese hamster ovary cells. As low as 0.31 mGy with alphas and 2.00 Gy for x-rays.
(45)	1995	Clinical bronchiolar lavage biochemistry	Patients receiving strictly unilateral radiotherapy for breast cancer were evaluated following postoperative radiotherapy.
(4)	1997	Clonogenic survival assay	Human epithelial cells. Medium transfer. Gamma source.
(5)	2009	Micronucleus assay	X-rays and alpha particle microbeam.

## Specific studies of DNA repair effects

Early ideas to explain the “How” question involved the work by Little’s lab (46, 47), and they found that gap junction intercellular communication (GJIC) was involved. Lindane was used as an inhibitor of GJIC and connexins involved in the junctions were shown to be upregulated in cultures of irradiated cells exposed to ultra-low fluences. However, while GJIC could explain the ultra-low fluence and microbeam experiments, it could not explain the medium transfer experiments. The most accepted explanation for these involved the generation of reactive oxygen species (ROS) and reactive nitrogen species (NOS), which are believed to be generated by the direct irradiation that induced secondary damage in the surrounding unirradiated cells (48–50). Sophisticated experiments using available microbeams at the Gray Laboratory in the UK or at Columbia University in the United States measured the spread of these from the targeted cell (51, 52). However, as ROS and NOS were short lived, they could not fully explain medium transfer experiments, where medium harvested hours after irradiation could induce both bystander effects and genomic instability in unirradiated recipients; this includes the induction of apoptosis, calcium release, mitochondrial membrane depolarization, and caspase release, which culminated in visible apoptosis (53–55). These experiments gave rise to the idea that soluble factors were released by irradiated cells and there was much discussion then about how big these factors were based on diffusion gradients from microbeam experiments. No “magic factors” were characterized, and in fact, some experiments by our group suggested that it was actually a loss of factors rather than active secretion of a molecular species that was the cause of RIBE. Experiments suggested that serotonin, naturally present in serum or added to culture medium, was critical and was bound by irradiated cells triggering downstream events including calcium release in recipients of the medium (56–61). There were also major advances in understanding the processes involved in both the targeted cells and the recipient cells. These mechanisms have been reviewed in detail many times—see, for example (29, 62, 63),—and involved activation of membrane receptors involved in calcium storage and release, serotonin binding, TGF beta and cytokine cascades, and activation of apoptosis. **Figure 1** shows currently accepted events occurring in targeted and non-targeted cells and **Table 2** lists these with key references. Because of the involvement of calcium and the apoptotic cascade, there was a considerable amount of work focusing on mitochondria and their role in DNA damage and repair in both targeted and non-targeted cells.

**Figure 1:**
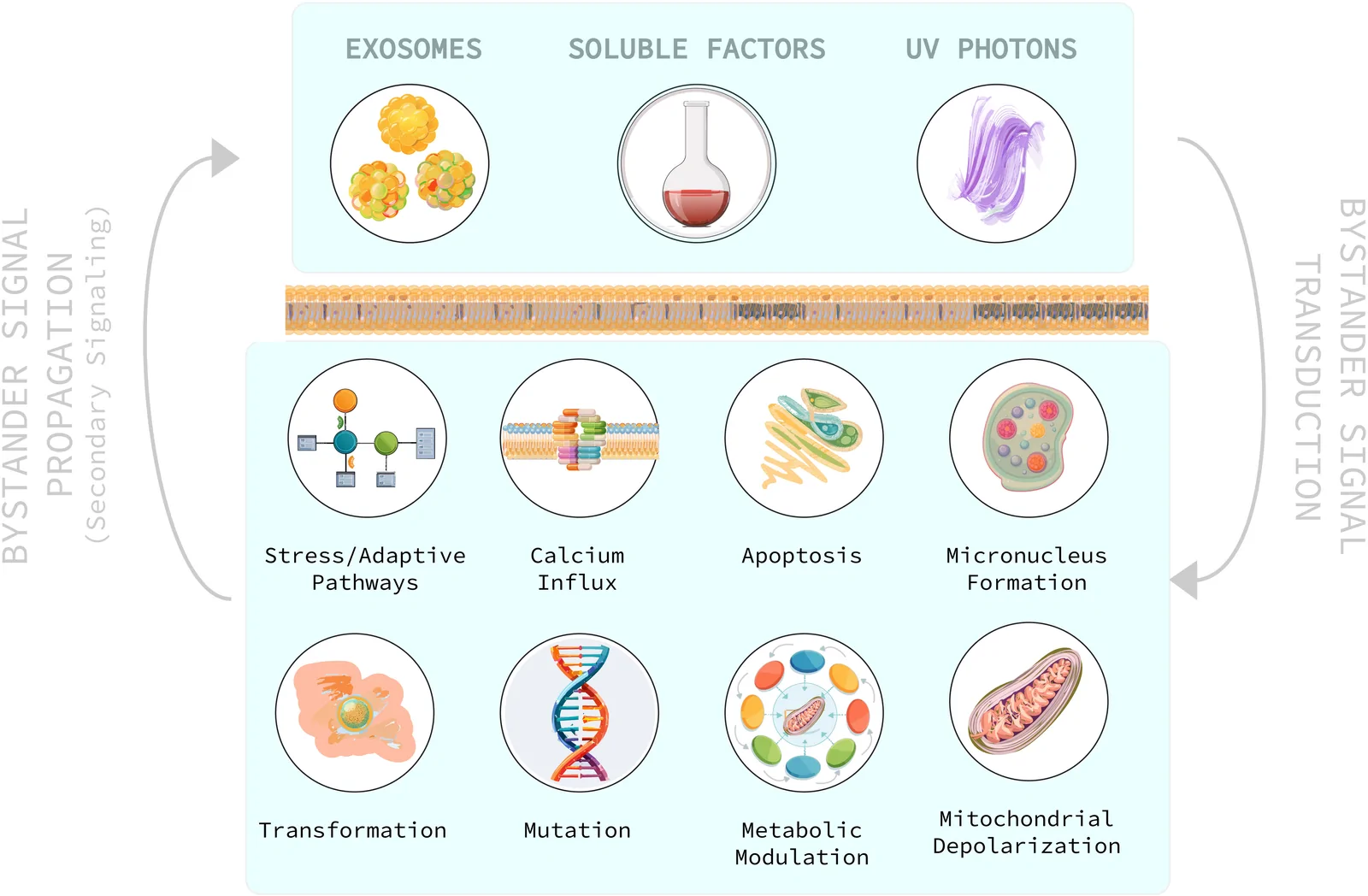
Currently accepted events occurring in targeted and non-targeted cells exposed to ionizing radiation. In directly irradiated cells, exosomes, soluble factors, and UV biophotons are released and convey exposure signals to surrounding cells. In bystander cells, various effects are produced in response to these signals.

**Table 2:** Key papers demonstrating currently accepted events occurring in targeted and non-targeted cells.

Ref.	Year	Journal	Effect	Notes
(49)	1996	*Carcinog.*	ICCM	Showed that radiation exposure could produce persistent oxidative stress and RIGI in bone marrow cultures. The work supported the idea that radiation effects can be delayed, self-propagating, and maintained through oxidative processes.
(46)	2001	*HJMS*	GJIC	Provided direct evidence that GJIC contributes to RIBE following α-particle irradiation. This identified cell-to-cell communication, rather than only soluble factors, as an important mechanism.
(47)	2003	*PNAS*	GJIC	Demonstrated that expression of connexin43, a major gap-junction protein, is highly responsive to ionizing radiation and environmental stress. The findings linked radiation responses to changes in intercellular communication pathways.
(51)	2005	*IJC*	Microbeam	Investigated communication between irradiated glioma cells and fibroblasts and showed that bystander signaling can occur between different cell types. This demonstrated that bystander effects can involve heterogeneous cell populations, relevant to real tissues.
(53)	2006	*Rad. Res.*	ICCM	Showed that calcium signaling plays an important role in modulating radiation-induced bystander responses between glioma and fibroblast cells. The results identified intracellular calcium fluxes as an important component of signal propagation.
(48)	2007	*JRR*	ICCM	Found that treatment of irradiated cells with DMSO suppressed bystander effects in neighboring CHO cells. This implicated reactive oxygen species (ROS) in generating or transmitting bystander signals.
(58)	2007	*Env. Res.*	ICCM	Demonstrated that radiation-induced bystander effects can be modified by signaling amines. The findings supported the hypothesis that biogenic amines may influence intercellular radiation signaling.
(57)	2009	*Dose Res.*	*In vivo*	Found that reserpine treatment in zebrafish prevented communication of radiation-induced bystander signals between fish. This provided *in vivo* evidence supporting a possible role for serotonin or related signaling amines.
(50)	2011	*IJRB*	ICCM	Demonstrated that ROS generated in irradiated cells can stimulate release of signaling factors that activate membrane signaling and calcium influx in bystander cells. This proposed a mechanistic chain involving ROS and calcium signaling.
(59)	2011	*IJRB*	ICCM	Found no significant influence of serotonin concentrations in fetal bovine serum on radiation-induced bystander effects or genomic instability. This challenged studies proposing a universal role for serotonin and suggested that its effects may be context-dependent or experimental system-dependent.
(56)	2012	*IJRB*	ICCM	Compared results between laboratories investigating whether serum serotonin affects radiation-induced bystander effects. The study highlighted substantial experimental variability and complexity in determining serotonin’s role.
(60)	2012	*IJRB*	ICCM	Examined the influence of serotonin and p53 status on radiation-induced bystander effects. The study suggested that cellular genetic background, particularly p53 signaling, can influence how cells respond to serotonin-associated bystander signaling.
(64)	2018	*Rad. Res.*	ICCM	Found that direct radiation responses and bystander effects can vary between cell lines depending partly on fetal bovine serum serotonin concentrations. The study helped reconcile conflicting earlier results by suggesting that serotonin’s effects are cell-type-dependent rather than universal.

## Mitochondrial DNA and mitochondrial involvement in NTE

The role of mitochondria has been reviewed very thoroughly by Averbeck in a major review (65) and by others (66, 67) who established the concept of DAMPs (damage-associated molecular pathways). This was done in an attempt to make sense of the accumulating data that almost everything measured in targeted cells could also be measured in non-targeted cells, and that mitochondria seemed to be key players in these processes (68, 69). Direct effects on mitochondrial DNA were measured by Nugent et al. (70). Lyng and colleagues reported mitochondrial membrane depolarization as a robust marker of bystander signaling (19, 71) and altered energy production was also reported (72, 73). A series of papers from our group then established a connection between mitochondrial complex 1 function and radiation-induced UVA biophoton emission from directly irradiated cells, which was proven to be both necessary and sufficient to produce bystander effects in unirradiated cells in separate flasks with no medium contact (74–76). Spectral emissions at other wavelengths and acoustic vibrations have also been detected from cells during irradiation, but these have not yet been definitively linked to bystander effects (77). In parallel with the biophoton emission work, another strand of research showed that irradiated cells release exosomes and other vesicles, which can induce bystander effects in non-targeted cells (78–80). These authors suggested that the exosomes contained information in the form of miRNAs that led to the downstream effects seen in the non-targeted population. To reconcile the two research hypotheses, Le et al. (81) performed experiments where exosomes were extracted from flasks of cells that had sat above flasks containing cells being irradiated with tritium. The beta tracks from the tritium in the donor flask could not have reached the cells atop these flasks (see **Figure 2** for a schematic of the experimental setup). The extracted and purified exosome fraction was then added to a completely separate set of cells and all sets of cells including controls were examined for evidence of mitochondrial damage or reproductive cell death. Both endpoints could be demonstrated in response to receipt of exosomes from the flasks that had received UVA biophotons or radiation but not in controls or in flasks where UVA was blocked using a yellow cellophane filter. These experiments clearly link ionizing radiation, UVA photon emission, and exosome release and demonstrate that exosome fractions from cells secondarily exposed to UVA photons can induce effects detectable using two endpoints associated with RIBE—mitochondrial membrane depolarization and reproductive cell death or mitotic death. In the early modern era of RIBE and RIGI research, which included work up to about 2005, the phenomena were considered to be solely adverse and to mean that risks from low-dose exposures to radiation were more dangerous than previously thought because they expanded the target area for possible radiation damage (82–84). However, more recent data suggest that this idea might be an oversimplification and that the view of NTE as increasing low-dose risk reflected more the endpoints being used rather than the true biology at play. The evidence for the more nuanced view is discussed below.

**Figure 2:**
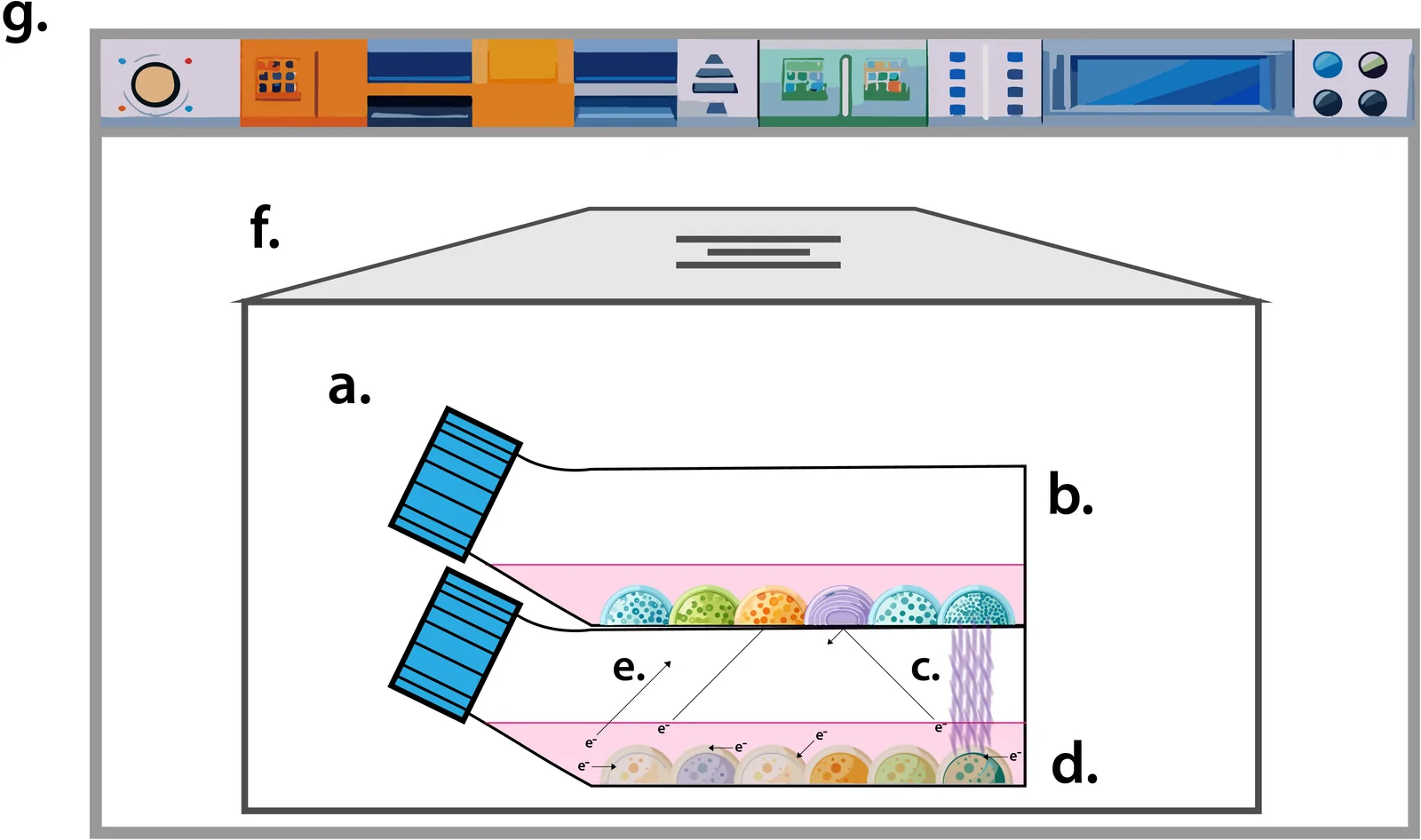
A simplified illustration of the experimental apparatus in Le et al. (53). Tissue culture flasks were stacked **(a)** with bystander reporters **(b)** situated above bystander reporters **(d)**. Detectable emissions of biophotons within the UV range **(c)** had been detected in cells exposed to beta radiation **(e)** from tritium in the donor flask. The flasks were placed in a light-tight metal enclosure **(f)** within a standard cell culture incubator **(g)** under normal conditions. Though most energy was absorbed by the growth medium and donor cells, even beta particles not attenuated sufficiently passing through medium and air cannot penetrate through the polystyrene; however, UV photons can pass from the donor flask to the recipient flask.

## Do NTEs lead to a protective (adaptive) response?

The first evidence that ARs might be induced in non-targeted cells came from the Columbia group (18). They showed induction of both ARs and bystander effects in C3H10T1/2 cells. Later work by Ryan et al. (85, 86) showed that medium from irradiated cells could induce an AR in cells subsequently exposed to actual ionizing radiation. These responses were found to be very dependent on cell line (87). The conclusion from this body of work suggested that induced repair of DNA damage was involved and that the NTE signals could act like the very small adapting doses used in the early AR experiments (88). The suggestion of induced repair was at the time considered somewhat heretical! In a field seen as unrelated at the time, however, inducible repair was the explanation for the phenomena first described by Joiner’s group and by Skov et al. (23, 89) as low-dose hypersensitivity.

## HRS/IRR data

Low-dose hypersensitivity (HRS) is generally associated with induced radioresistance (IRR) and together they describe a type of semi-log survival curve with a steep dose response in the dose range below about 100 mGy, followed by a plateau and a change to a shallower dose–response curve at doses above about 0.5 Gy (23, 89–91). **Figure 3** gives a sample curve of this type, which shows the general shape but does not contain the actual data referenced in the figure legend. The change in dose response was attributed to there being a threshold dose above which repair of DNA damage was inducible (23). This was linked by Joiner’s group to PARP and ATR signaling (92, 93). Most of the cell lines showing HRS/IRR patterns of dose response were derived from gliomas, and as our group had suggested that tumor-derived cell lines that had resistant low-dose response phenotypes did not show bystander effects (94), we decided to collaborate with Joiner’s group to test their cell lines for RIBE. Initial experiments suggested that the glioma cell lines did not show RIBE, and an early suggestion (26, 90, 95) was that RIBE and HRS/IRR are mutually exclusive phenomena. However, more detailed investigation showed a much more intriguing pattern. In the HRS region of the dose–response curve, RIBE *could* be demonstrated. This was measured as a reduction in clonogenic survival following medium transfer, but when the dose applied in the bystander assay was in the IRR dose range (above 0.5 Gy), no RIBE was detected (96). This suggested that RIBE was a low-dose phenomenon that could be neutralized if DNA repair was induced. It also suggested that RIBE might be at least part of the mechanism accounting for the “shoulder” region of the semi-log survival curve. The shoulder was variously attributed to the capacity of cells to accumulate “sublethal DNA damage” or to repair “potentially lethal DNA damage”. For a recent review of this history, see (97). In the early work on RIGI/RIBE, the shoulder had been linked to RIGI and could be removed if a correction for delayed lethal mutations were applied to the classic Puck and Marcus survival curve (98, 99). Again, it must be stressed that these experiments were all measuring changes in the reproductive survival of irradiated cells at different population doublings post irradiation.

**Figure 3:**
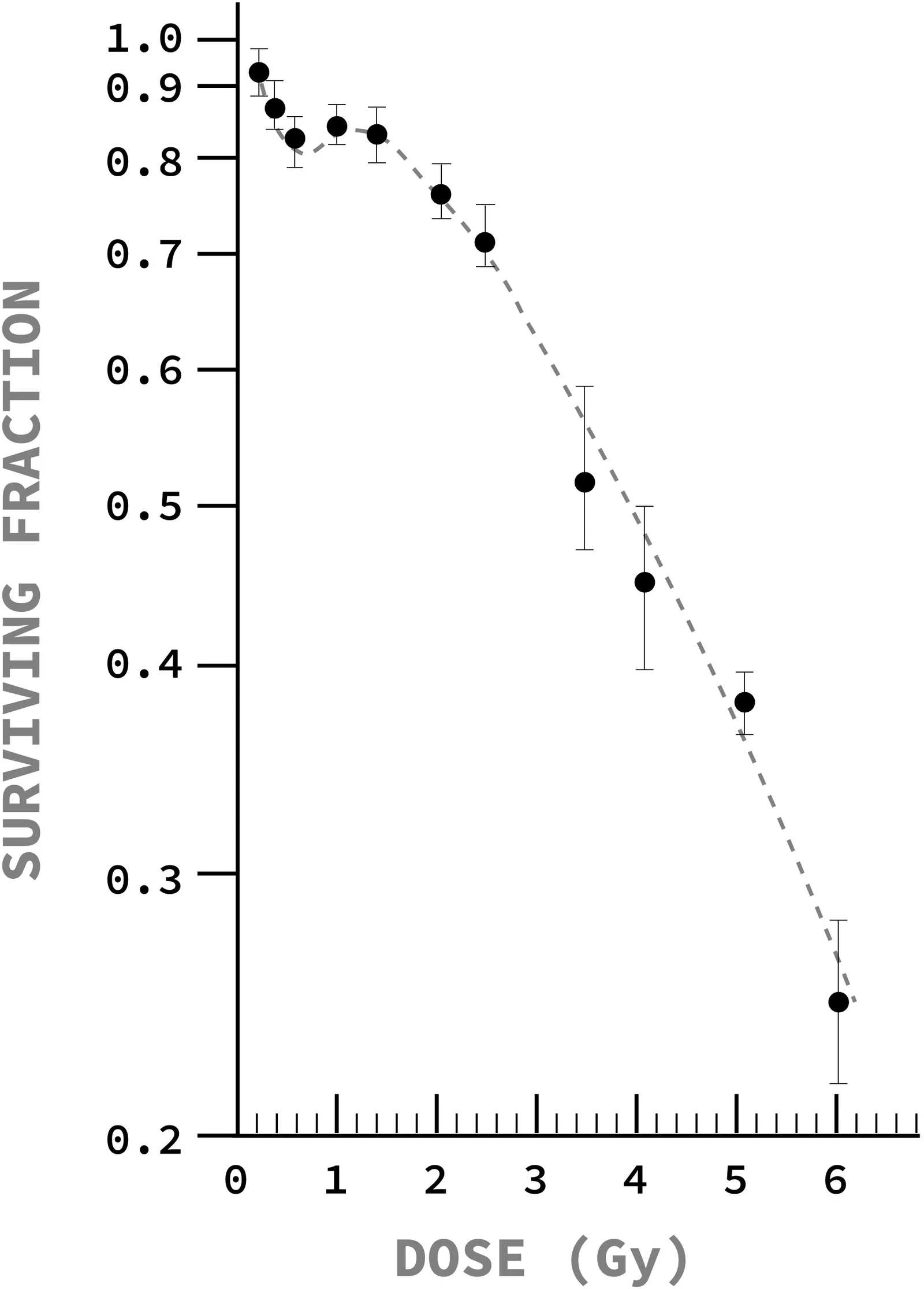
A generalized survival curve illustrating low-dose hypersensitivity (HRS) based on data from Joiner’s group (23). This figure demonstrates the general observed shape but does not contain actual data.

## Dose and dose rate effects

Key questions concerning the relevance of NTE for radiotherapy and radiation carcinogenesis required information concerning dose and dose rate effects. Experiments by our group and by Prise’s group determined that NTEs were triggered by very low doses in the region of 2–3 mGy of low LET radiation (100, 101). Interestingly, the effects seemed to saturate at doses above 0.5 Gy. A survival curve constructed over seven log cycles of dose [reconstructed in **Figure 4** from data generated by Liu et al. (102)] showed three distinct phases. Phase 1 up to 2-mGy dose showed no RIBE with a trend (not statistically significant) for the cells receiving medium transfer to have slightly higher than the control clonogenic survival. Survival values greater than 100% occur when the plating efficiency control value is less than the plating efficiency seen in the treated group. Phase 2 from 2 to 500 mGy showed no difference, whether cells were directly irradiated or received medium from irradiated cells. Phase 3 showed a clear divergence where the directly irradiated cells showed a classic radiation dose response and the cells receiving medium transfer showed no further increase in reproductive death. This suggested that at least for the cell line used (human keratinocytes), the RIBE effect was associated with low doses, had a very low-dose trigger, and saturated at about 0.5 Gy. This means that NTEs probably relate to DNA damage response mechanisms after low doses but may not be relevant to high-dose damage responses. Support for this idea also shown in **Figure 4** is the pattern of the calcium signal. A calcium flux resulting in a transient spike in intracellular calcium was seen by Lyng et al. (25) as a first sign of bystander signaling in cells receiving irradiated cell conditioned medium (ICCM). In Liu’s study, the calcium spike was detected only when using ICCM generated from irradiated cells receiving doses of 2 mGy or more. Doses below 2 mGy did not result in a calcium signal, indicating a mechanistic shift approximately 2 mGy for this cell line.

**Figure 4:**
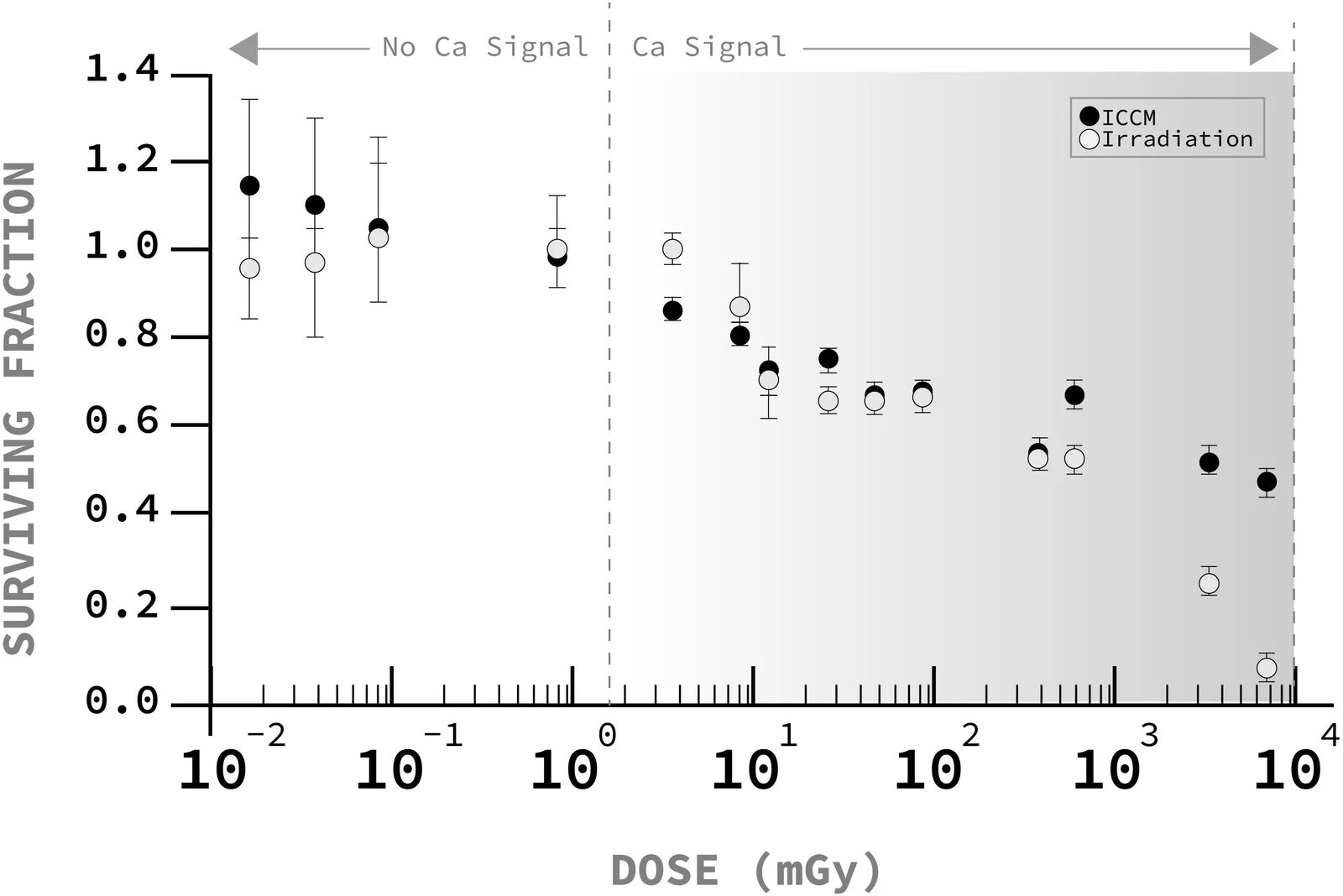
A reconstruction of the data generated by Liu et al. (102). Open symbols are clonogenic survival data for directly irradiated cells. Closed (black) symbols represent clonogenic survival curve data for cells plated as single cells then treated after 6 h with irradiated cell conditioned medium (ICCM) harvested from irradiated cells. Calcium release in response to addition of medium was measured for each ICCM dose point. “No ca signal” indicates that no calcium signal was seen. “ca signal” indicates a calcium signal was seen. Full details are in Liu et al. (102).

Dose rate effects remain unclear. Traditionally, *in vitro*, dose rate was studied using fractionation protocols that were then integrated to produce cumulative dose–response curves (103). The general conclusion was that doses delivered over protracted time intervals led to higher cell survival as repair could take place and repair capacity was not depleted. Studies of NTE in these situations showed that medium from irradiated cells could replace actual dose in some fractionated protocols, and this was interpreted to mean that repair was induced by signals in the medium (104). Some studies compared radiotherapy machines giving different dose rates or varied the source to target distance, but variations were small and major effects of dose rate were not reported (105). However, there are two areas where dose rate in the context of NTE and DNA repair could be relevant—one is in FLASH protocols using parallel microbeams, which use ultra-high-dose rates generated using synchrotron beams. The studies in this area report sparing of normal tissues, which is variously attributed to vaporization of oxygen or to bystander effects in the valleys between the microbeams (106–109). The other area is in the natural environment where low chronic doses are of concern (109). In the context of the topic under discussion, there are insufficient studies so far of NTE and DNA damage response in these situations.

## Radiation quality effects

A considerable amount of work has been done to determine if bystander effects occur with different radiation qualities. The data are summarized in **Table 3** together with key references. It is clear that alpha particles and low LET x- and gamma-rays all produce both bystander effects and genomic instability including lethal mutations. Work done by Held and colleagues using the transwell system and a variety of proton beams conclude that there is no difference in the RIBR between low LET x-rays or proton beams, at least in their system using osteoclasts (117, 118). The endpoints used included 53bp1, which measures repair of DNA damage, and micronucleus frequency in binucleated cells, which is a measure of division failure in cells. Other experiments using proton microbeams by Autsavapromporn et al. (107) showed RIBE in normal and in hypoxic tumor cells, but they were using FLASH protocols so the doses were high (about 10 Gy). It is known that proton beams can produce low levels of secondary gamma radiation, so given the very low threshold for triggering RIBE, it is not easy to determine if protons *per se* produced the observed effects.

**Table 3:** Summarized key radiation quality effects data.

Ref.	Year	Rad.	Notes
(98)	1988	Gamma, neutron	This earlier theoretical/experimental work examined lethal mutations resulting from the incorrect repair of radiation-induced lesions, termed Q-lesions. It contributed to the conceptual basis for understanding how radiation damage can lead to delayed lethal or mutagenic consequences through DNA misrepair.
(110)	1995	X-ray, alpha	This study demonstrated delayed chromosomal aberrations and apoptosis in primary human bone marrow cells after radiation exposure. It provided important evidence for radiation-induced genomic instability, showing that biological damage can emerge in descendant cells long after the original irradiation event.
(102)	2006	Gamma	The authors investigated whether there was a dose threshold for ICCM bystander effects in a human skin cell line. Their findings suggested that detectable bystander signaling was not necessarily induced at arbitrarily low doses, supporting the possibility of a threshold for generating sufficient extracellular damage signals.
(111)	2009	X-ray, neutron, alpha	Reviewed manifestations and mechanisms of radiation-induced genomic instability in V79 Chinese hamster cells. It emphasized that radiation effects can persist through multiple cell generations, producing delayed chromosomal abnormalities and other genomic changes that cannot be explained solely by the initial DNA damage.
(112)	2011	Neutron	Using zebrafish irradiated *in vivo*, the authors found no evidence of a neutron-induced bystander effect under their experimental conditions. This important negative result suggests that bystander effects are not universally produced by all radiation qualities or biological systems.
(113)	2014	Neutron	Examined neutron exposure in human cells, assessing both direct biological effectiveness and bystander effects. The results suggested that neutron radiation has high biological effectiveness, but that bystander responses must be considered separately from direct neutron-induced damage when evaluating overall biological risk.
(114)	2019	Neutron	This comprehensive review investigated whether very low neutron exposures could induce radiation bystander effects. It addressed the lower-dose limits of neutron-induced signaling and contributed to the debate over whether neutron radiation can reliably generate bystander responses at environmentally or biologically low exposures.
(115)	2021	Gamma, neutron	This study examined the DNA damage response of hematopoietic stem and progenitor cells following high-LET neutron irradiation. It demonstrated that neutron exposure produces substantial and complex DNA damage responses in these radiosensitive cells, with implications for bone marrow injury and long-term genomic consequences.
(107)	2026	Proton	Examined RIBE in hypoxic lung cancer cells and normal lung fibroblasts exposed to conventional versus ultra-high-dose-rate (FLASH) proton microbeam irradiation. FLASH irradiation produced a relative protective/sparing effect in bystander cells, particularly normal cells, with gap-junction communication and antioxidant responses, including possible differences in Nrf2 activity, implicated in the response.
(116)	2026	Neutron	This computational study used track-structure simulations and DNA repair modelling to estimate neutron relative biological effectiveness (RBE) before and after DNA repair. It highlights how neutron RBE depends not only on initial DNA damage complexity but also on subsequent repair and misrepair processes.

Similarly, the question of whether neutrons produce RIBE/RIBR is complicated because most beams have gamma contamination, as reviewed in (114, 119). Work by our group using the Gray Laboratory beam, which had only 6% gamma contribution, showed no RIGI (measured via lethal mutations) with neutrons (98); however, work by Trott’s group performed using the Gray laboratory source showed genomic instability after 2.5 and 5 Gy neutron exposure (111). Work by our group using the McMaster nuclear reactor beamline (thermal neutrons) determined that, if the gamma component was shielded out, no RIBE was seen, although the doses here were much lower at approximately 100 mGy (102). Further experiments (Liu et al., abstract accepted for EPRBioDos conference in Montreal September 2026) suggest that there is some evidence, using fast neutrons generated by the Tandetron accelerator at McMaster University, that neutrons may actually neutralize the gamma effect in the cell lines used (skin keratinocytes and p53 wild-type HCT-116 cells), as there was no detectable bystander effect of the gamma component of the beam. Also, another study using a fast neutron beam with approximately 5% photon dose contamination similarly reported no RIBE following neutron irradiation, with no detectable effect attributable to the photon contamination (113). That paper used micronucleus as endpoint. Other evidence that neutrons *per se* do not produce RIBE comes from an experiment using the fish–fish communication system (112). Using this system, robust gamma RIBE was detected, but no effects were seen with neutrons. Regarding DNA repair endpoints in relation to neutron RIBE, we did not find any studies that directly investigated DNA repair endpoints in a neutron bystander context. There are only a few studies looking at DNA repair endpoints following direct neutron irradiation (115, 116).

Our conclusion at this point is that ionizing radiation of any quality can produce NTE but low doses of “pure” neutrons do not produce NTE. High doses of pure neutrons can produce NTE indirectly due to emission of charged particles and secondary ionizations.

## *In vivo* demonstrations of NTE

Most of the early demonstrations of NTE were done *in vitro* using cell lines or, in some cases, primary cultures, such as the bone marrow cultures used by Wright’s group in the UK that permitted chromosomal aberrations in clonal lineages to be demonstrated as non-clonal aberrations (110). The widespread use of cell lines caused critics to suggest that NTEs were just artifacts of cell culture and not relevant for health of humans or non-humans. This was even though the pre-modern era studies up to the 1970s actually were mostly done using blood or tissues from *in vivo* exposed radiotherapy patients or experimental animals (13). Now, however, there is ample evidence from multiple studies that NTEs occur *in vivo*. Studies by Watson et al. and Zhao et al. demonstrated RIGI in bone marrow from irradiated mice (120, 121). These studies demonstrated, using a cross sex bone marrow transplant in mice, that transplanted cells could induce aberrations in regenerating marrow from the opposite. Studies of abscopal effects in lung tissue also showed *in vivo* evidence of RIBE (122) and further studies by Demaria and others suggested an involvement of the immune system in the mechanisms [reviewed in (29, 123)]. This was also demonstrated by Wright’s group who linked NTE to inflammatory responses (124). Bystander effects were also found in the serum taken from Chernobyl-affected populations as long as 20 years after the reactor explosion, and genomic instability was detected in multiple generations of bank voles descended from exposed populations in the Belorussian Polessky Radiation and Ecological Reserve (PGREZ) (125, 126). This suggested that NTEs are induced and persist over multiple generations and for many years after exposure. Work by Surinov and later by Daev in Russia demonstrated that irradiated mice could produce volatile signals that caused chromosomal damage in unirradiated littermates (127–129). These intriguing findings led our group to look at inter-animal signaling in fish (see **Figure 5** for our general experimental protocol). In a series of papers, we demonstrated this using rainbow trout, zebrafish, and in a strain of DNA repair-deficient medaka fish. Fish were irradiated and placed in an aquarium with unirradiated fish separated by a mesh that allowed water to circulate but permitted no physical contact. The irradiated fish were shown, using a reporter system, to have transmitted bystander signals to the unirradiated fish (7, 130). Follow-up proteomic analysis revealed that the bystander fish had induced a “protective” proteome with antioxidant and DNA repair proteins among the changes from controls (131, 132). The directly irradiated fish conversely did not show this protective proteome. The series of fish experiments also provided the first evidence that biophotons or acoustic signals might be involved in the communication mechanism (133). Later experiments with earthworms, crickets, and mice confirmed inter-animal signaling as a general phenomenon (134–136). Many of the methods for demonstrating *in vivo* NTE involve *in vitro* reporter systems, such as explanted pieces of tissue, upon which the assays are performed, but experiments by Li et al. (134) using crickets and by Dudley’s group using plants demonstrated direct effects of irradiated organisms on neighbors resulting in morphological effects. Dudley’s experiments suggested the involvement of mycelium (137) in these effects. The bottom line is that there is increasing evidence of NTE *in vitro* and *in vivo*. The mechanisms and endpoints so far elucidated suggest considerable epigenetic and post-translational modification of responses in neighbors affected by signals; thus, the questions for oncology researchers are to understand not just the mechanisms but also the consequences of this signaling and communication process. Clearly at the practical level, there may be opportunities for identifying novel targets for therapy, but at the treatment planning level, there are also questions regarding how out-of-field and abscopal effects might play out during treatment. In the field of radiation carcinogenesis, there are also issues for radiation protection of humans and of the environment. Some of these are discussed in the next section of this paper.

**Figure 5:**
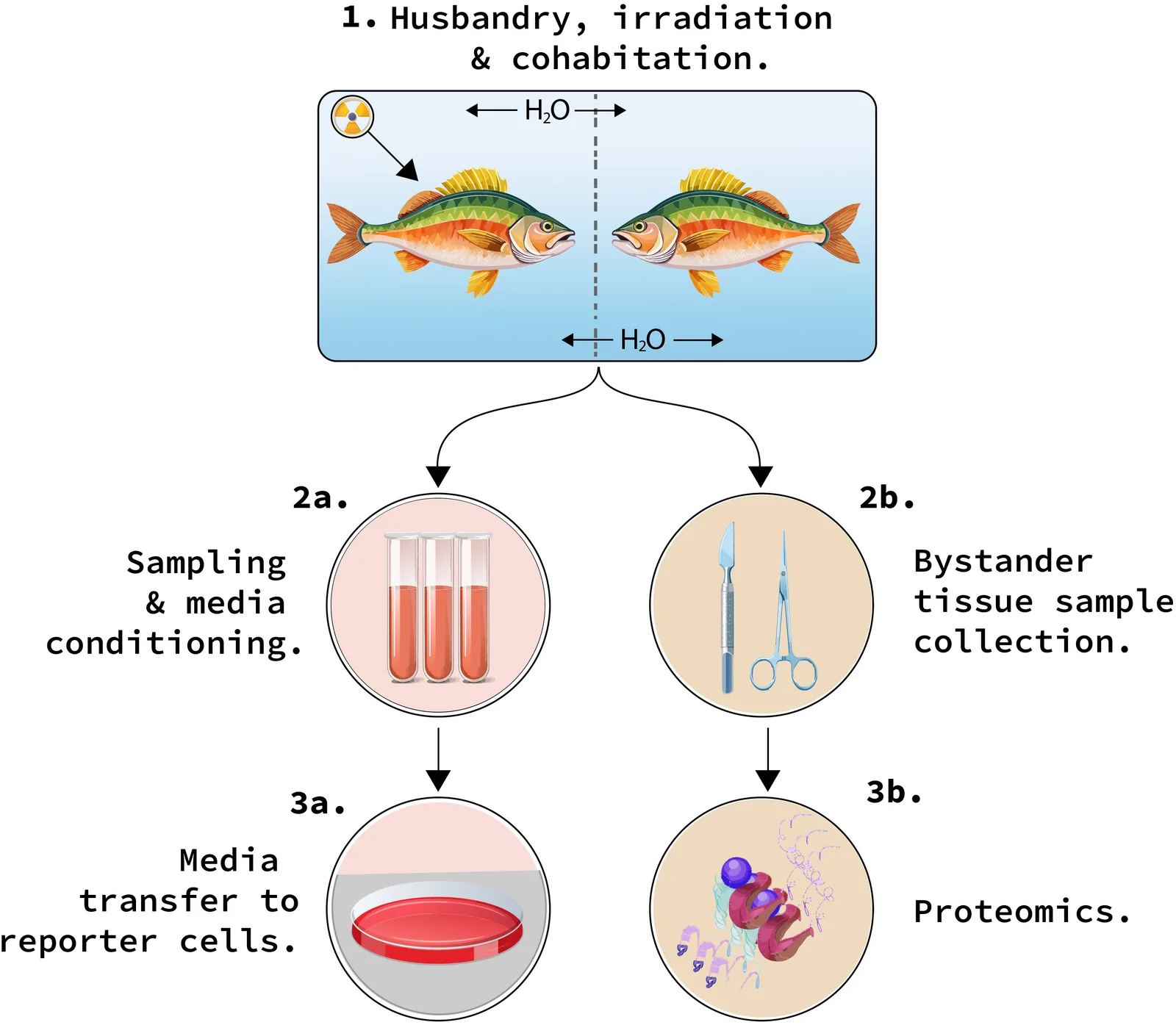
A general protocol for the fish work in (7, 130–132) that demonstrated non-targeted effects including RIBE *in vivo*.

## Discussion

### What do targets mean now?

Classical radiobiology is built on target theory (138, 139), where radiation interactions with living cells were analyzed in terms of the number of hits needed to “inactivate” the cell (generally taken to mean “to prevent cell division”). As our knowledge about DNA structure, location, and function increased, so did the formalism of target theory. As tools evolved for looking at DNA damage and for precise dosimetry and microdosimetry, so the tenets of target theory became more precise and various survival curve models were advanced to explain the dose–response curve. This had an enigmatic low-dose shoulder in conventional semi-log plots, which actually encompassed up to 90% of the cell inactivation (140). In defense of the system, it was developed to understand how radiation worked to kill tumor cells in radiotherapy, where killing every last tumor cell while leaving tissue function as good as possible was the goal. All the odd results that did not fit were largely ignored. These results were recently reviewed by these authors (97). Really important results challenging accepted standards such as those used to justify the use of fractionation (iso-effect per fraction) were also ignored despite mounting evidence of ARs and field effects (139). The paradigm shift that occurred in the late 1980s and early 1990s was partly due to the availability of tools to look at low doses, but it was also due to the influx of biologists into radiobiology who considered the idea of targets as unique entities to be inactivated as rather strange. In biology at the time, concepts such as programmed cell death (apoptosis), inducible repair, ARs, and other mechanistic responses that were established and accepted were not compatible with the rigid formalism in radiation physics of passive target inactivation. Targets as a concept are still used but their meaning is completely different, referring to molecular targets for drug development or key drivers of biological response to an insult. Even the use of the term “non-targeted effects” is probably inaccurate. It was coined during the European Union’s RADINSTAB project as an attempt to gather all the disparate low-dose effects under one umbrella. The NOTE (**NO**n-DNA-**T**argeted **E**ffects) project was the result. Modern interpretations of NTE regard them as part of a suite of communication and signaling mechanisms.

### What is the role of signaling and communication in radiation carcinogenesis and therapy?

Cell signaling and communication is a huge area of research and involves immune and endocrine signaling, microbiome signaling, and other pathways that are outside the scope of this review. The discussion here is restricted to NTEs resulting from signaling from irradiated to non-irradiated cells and what they might mean for our current theories about radiation carcinogenesis and radiotherapy. Critical to this discussion for both carcinogenesis and therapy is a consideration of the differences in bystander cell responses in normal versus tumor cell populations, in *ex vivo* explanted tumors and normal tissues, and in animal models. Early studies of RIBE suggested that they did not occur or sometimes led to increased reproductive survival in tumor cell lines (141, 142). There were exceptions such as the SW48 highly radiosensitive cell line derived from a colon carcinoma and more detailed investigation led to the conclusion that the p53 status of the cell line might determine whether bystander effects were seen or not (76, 143, 144). Cell lines with wild-type or null p53 expression had RIBE, but in general, mutant p53-expressing cell lines did not show effects. Later mix match experiments tried to resolve whether the signal production or the response to the signal were critical, but these did not give a conclusive answer, suggesting that other factors are involved in RIBE expression. Recent preliminary data from McQuoid (PhD thesis, Queen’s University Belfast) (personal communication) using TP53 mutant FaDu cells (HNSCC), which have a single hotspot mutation R248L, and DU145 cells (PCa), which have two missense mutations P223L and V274F, do show RIBE using clonogenic survival and micronucleus formation; thus, clearly more work is needed to resolve this question. It may be that radiosensitivity *per se* is the critical factor rather than any specific gene product. Support for this idea comes from work by our group and by Joiner’s group (145–148) linking expression of genomic instability, HRS, and bystander effects to the DNA repair capacity of the cell line. Mothersill et al. (146, 147) showed that, if NTEs were measured in wild-type parents and derived repair-deficient mutant cell lines, the reduction in clonogenic cell survival in cultures receiving bystander medium was greater in the repair-deficient mutants than in the parent cell lines. Zhao et al. (148) showed a correlation between the alpha/beta ratio and the occurrence of the HRS/IRR pattern of survival, and our groups collaborated to show that the pattern of RIBE was associated with the HRS part of the dose–response curve as mentioned above. These data together with the p53 studies suggest that radiosensitivity and the ability to repair DNA may be key to preventing RIBE-associated reductions in clonogenic cell survival. However, this is like “looking under the lamppost”, and it is likely that signal production occurs quite commonly although not universally (see the “Radiation quality effects” section above) but is interpreted differently in the cells receiving signals depending on prevailing micro- or macro-environmental factors. For example, hypoxic tumor cells may not have pathways activated to allow apoptosis, or lactate in tumors may modulate response to signals. Some evidence for this was published in 2000 by our group in collaboration with Tom Stamato (72). This paper showed a role for energy metabolism in generating NTE and considered Warburg biochemistry as being critical. In today’s world, where there is a much more holistic vision of low radiation dose effects including NTE, this is not surprising, and it fits with the response-driven approach to radiation biology where concepts such as resilience and energy budgets are considered important in determining the outcome.

### What is the role of exosomes?

The release of exosomes (then simply called cellular vesicles) was first noted as a possible radiation response by Albanese and Dainiak in 1982 (149). That research remained relatively obscure until a paper by Kadhim’s group in 2012 (150) showed release of exosomes from irradiated cells that contained “information” that induced bystander responses in non-targeted cells. This was subsequently confirmed by Lyng’s group in Dublin (151). Exosomes and RIBE were the subject of an EU project that resulted in a comprehensive publication detailing the NTE mechanisms in relation to exosome structure and function (152). Exosomes have also been studied by Ariyoshi et al. in relation to NTE (78). These authors determined that mitochondrial DNA in the exosomes communicated information to bystander cells. The research findings surrounding exosomes and other signals are discussed above, but here, it is important to consider their role in relation to DNA damage and repair in non-targeted cells. The larger question to be addressed in the final section is whether they are relevant for therapy or radiation protection.

### What is the role of photon and phonon emissions?

We have known about ultralow photon emissions since the 1920s [reviewed in (153)], but consideration of emissions as contributing to NTE only started in 2013. Acoustic emissions were known in the plant world, but relevance in human medicine had not been seriously considered. Now, the question is whether the emissions have any clinical implications. The clear evidence of UVA photon emissions from irradiated cells and the documented mitochondrial effects suggest that they could reduce energy production, thus limiting DNA repair potential in the targeted field and also in bystander cells outside the targeted field. There is also the possibility of UVA damage in internal organs in the radiation field, which would not normally be exposed to UV. It might be interesting to measure specific UV repair enzymes in tissues in the ionizing radiation beam. Bystander effects *in vitro* could be stopped using yellow cellophane or using black-coated multiwell plates; thus, would UV absorbing materials change therapeutic outcomes?

### Implications

While the existence of NTE is no longer in doubt, there is no consensus on whether they have any relevance for either radiation protection or for imaging and radiotherapy. This is surprising since they are low-dose effects with clearly demonstrated effects on energy production, mitochondrial function, DNA damage, and repair (which is energy dependent). Some possible implications are considered below in the final section of this narrative review.

### Implications for treatment and diagnostic imaging?

Key considerations here are the out-of-field effects or abscopal effects caused by bystander signaling. The questions are whether the signals could be exploited to improve therapy or to better “map” tumors in the field of diagnostic imaging. The recent growth in imaging the tumor ecosystem using suites of immunohistochemical markers (154) would suggest that identification of cells producing signals would be feasible as would identification of target cells receiving the signals. Even if the actual tumor cells are not secreting signals, their support network of fibroblasts, immune system cells, or endothelial support blood vessels might be. Better information about the tumor ecosystem in relation to NTE might yield novel biomarkers or therapeutic avenues; for example, these may be based on mitochondrial metabolism or the apoptotic cascade. Serotonin receptors might also be useful as discussed earlier.

### Implications for radiation protection?

The implications for radiation protection of the environment have been discussed in detail in a series of workshop reports involving the International Union of Radioecologists [see, for example (155)]. These make the case that NTEs are relevant for low-dose and chronic exposures because they involve signaling between and within populations of species in ecosystems. They are seen as important in adaptation and evolution of populations and may coordinate population-level responses. While much of the discussion is speculative given the difficulty of identifying low-dose radiation effects in complex environments with multiple stressors, there are papers discussed earlier using theoretical models, animal models, and field work that document both bystander effects and genomic instability in biota. The latter is seen in the Belorussian studies referred to earlier as multigenerational expression of chromosomal instability in voles monitored for 20 years after the accident or of clastogenic factors persisting in serum from Chornobyl-exposed human subjects many years after exposure. Both datasets documented persistent DNA damage endpoints. Key concepts include resilience, adaptation, and historic dose.

In the human radiation protection field, the goals are different. They involve protection of individuals from cancer rather than protection of populations or ecosystems from collapse. Here, there is limited discussion of NTE as they are regarded as part of the uncertainty surrounding low-dose radiation response. BEIR and UNSCEAR reports merely suggest that no conclusions can be drawn about the relevance of NTE at this time (156, 157).

## Conclusion

In conclusion, it is likely that the fields of imaging and therapy would both benefit from greater mechanistic understanding of NTE and they are likely to be highly relevant mechanisms in population response to low-dose radiation exposure at the level of cell, tissue, and organism communication and signaling.
